# Probing Cortical Activity During Head-Fixed Behavior

**DOI:** 10.3389/fnmol.2020.00030

**Published:** 2020-02-28

**Authors:** Ann-Sofie Bjerre, Lucy M. Palmer

**Affiliations:** Florey Institute of Neuroscience and Mental Health, University of Melbourne, Parkville, VIC, Australia

**Keywords:** cortex, head-fixed, sensory-based behavior, primary sensory cortices, posterior parietal cortex, anterior lateral motor cortex, Go/NoGo, 2AFC

## Abstract

The cortex is crucial for many behaviors, ranging from sensory-based behaviors to working memory and social behaviors. To gain an in-depth understanding of the contribution to these behaviors, cellular and sub-cellular recordings from both individual and populations of cortical neurons are vital. However, techniques allowing such recordings, such as two-photon imaging and whole-cell electrophysiology, require absolute stability of the head, a requirement not often fulfilled in freely moving animals. Here, we review and compare behavioral paradigms that have been developed and adapted for the head-fixed preparation, which together offer the needed stability for live recordings of neural activity in behaving animals. We also review how the head-fixed preparation has been used to explore the function of primary sensory cortices, posterior parietal cortex (PPC) and anterior lateral motor (ALM) cortex in sensory-based behavioral tasks, while also discussing the considerations of performing such recordings. Overall, this review highlights the head-fixed preparation as allowing in-depth investigation into the neural activity underlying behaviors by providing highly controllable settings for precise stimuli presentation which can be combined with behavioral paradigms ranging from simple sensory detection tasks to complex, cross-modal, memory-guided decision-making tasks.

## Introduction

Our behavior, the way one acts or conducts oneself, is key to survival. Animals must behave in an appropriate manner to successfully navigate and interact with their surroundings. This involves billions of neurons working together to formulate a cohesive motor output. Therefore, due simply to the sheer numbers involved, understanding the neural basis of behavior is complicated. It is further confounded by the diversity of behaviors, ranging from perception to social interactions to navigation, which involve different classes of neurons, neural interactions as well as brain regions. The activity of individual neurons within the cortex are often highlighted as being crucial for many behaviors, such as sensory-based (Xu et al., 2012; Takahashi et al., 2016) and social behaviors (Rao et al., 2014; Lenschow and Brecht, 2015), as well as anticipation (Erlich et al., 2011; Guo et al., 2014b) and decision making (Harvey et al., 2012). However, it is difficult to clearly state the overall role of the cortex, or a class of cortical neurons, during behavior as the underlying neural activity is dependent on many factors, such as changes in feedforward and feedback information, as well as overall brain state (Poulet and Petersen, 2008).

To understand the contribution of individual neurons during behavior, recordings must be performed from an individual or population of identified neurons, during an active behavioral task. Recording action potential firing in a population of neurons in freely-behaving rodents can be achieved using extracellular probes with a high density of recording sites (Buzsáki, 2004). However, extracellular recordings are fraught with analysis considerations (Buzsáki et al., 2012; Higley, 2012) and cannot typically measure sub-threshold and sub-cellular activity (although see Suzuki and Larkum, 2017). The mere act of behaving makes recording the activity of individual neurons challenging, as most techniques used to measure the activity of individual neurons, such as calcium imaging and patch-clamp electrophysiology, requires absolute stability of the recording preparation. Although miniaturized equipment can record sub-cellular calcium (Helmchen et al., 2001; Ghosh et al., 2011; Cai et al., 2016) and voltage (Burgalossi et al., 2011; Tang et al., 2014) activity from single neurons in animals as they physically move through an environment, these are highly specialized and difficult to implement. Therefore, most studies delving into the sub-cellular neural activity associated with behavior are performed in the head-fixed condition. Unlike the freely moving animal models, the head-fixed preparation allows for highly controllable settings, such as stimulus presentation and motor output. Whether it requires precise stimuli for studying perception, discrimination, decision making or other cognitive tasks, the head-fixed preparation provides the opportunity to precisely control the stimulus delivered to the animal. Head-fixed behavioral models also typically provide high repeatability and employment of standardized techniques (Guo et al., 2014a). This review article brings together studies investigating the neural activity during various behaviors developed for head-fixed preparation. Here, we focus primarily on the role of the cortex in sensory-based behaviors. Historically, research into the role of the cortex during sensory perception and decision making was typically performed in primates (for review see Parker and Newsome, 1998), however, due to recent advances in molecular and optical techniques, rodents are now a crucial animal model and will be the focus of this review.

## Behavioral Paradigms

Since there is no one-fits-all behavioral model or task that will highlight the role of all brain regions, it is crucial to consider which model or task will be the most appropriate to engage a particular brain region and how to pair this with an appropriate recording technique. In the head-fixed preparation, the stimulus typically needs to be brought to the animal, as opposed to the animal moving to the stimulus in the freely moving scenario. Therefore, various behavioral tasks have been specifically developed for the head-fixed preparation. These tasks range in complexity, from presenting specific sensory stimuli to the animals to reconstructing an entire sensory environment.

### Go/NoGo

**Figure 1:**
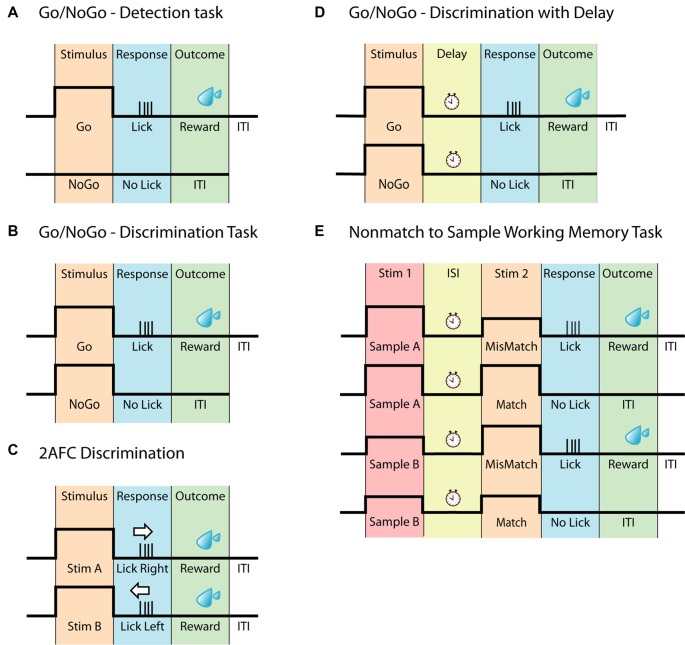
Head-fixed behavioral paradigms. **(A)** Schematic of the Go/NoGo task often referred to as a detection task. The animal should only respond upon detecting the stimulus. **(B)** Schematic of the Go/NoGo task involving discrimination of two stimuli. The animal should only respond to the Go-stimulus. This task is often referred to as a discrimination task. **(C)** Schematic of the two-alternative forced-choice (2AFC) task. Similar to **(B)**, this task requires the discrimination between two different stimuli however, to eliminate pseudo responses, the animal must respond to both stimuli, for example by either licking left or licking right. **(D)** Schematic of the Go/NoGo discrimination task with delay. This task differs from **(B)** by having a delay separating the stimulus and the response. **(E)** Schematic of the Nonmatch to Sample Working memory task, based on the Go/NoGo discrimination paradigm. In this task, the animal is presented with two consecutive stimuli, separated by a delay. The animal must compare the two stimuli and respond accordingly.

Perhaps the easiest way to explore perception and decision making is by presenting one stimulus associated with a single response. This is the basis of the Go/NoGo task (Figure 1A), where an animal is presented with a stimulus and trained to report the perception of the stimulus, by either responding or withholding an action. This creates a Hit/Miss scenario for Go trials and a Correct Rejection/False Alarm scenario for NoGo trials. The Go/NoGo task can be used to address simple questions such as sensory perception and modulation (Petreanu et al., 2012; Xu et al., 2012; Takahashi et al., 2016; Micallef et al., 2017). In general, this paradigm takes only a few days to implement (1–3 days; Guo et al., 2014a; Micallef et al., 2017), however, a common weakness of the task, is a potential deviation in motivation leading to erroneous reporting. To overcome the decreased motivation towards obtaining a reward, the Go/NoGo task can be further advanced by applying the signal detection theory, as reviewed by Carandini and Churchland, (2013). By introducing a stimulus-based NoGo signal that is of same stimulus type but of different character (rather than the absence of a stimulus as a NoGo signal, commonly referred to as “Catch trials”), the animal has to discriminate between two stimuli, reporting only one of them, instead of simply reporting whenever the stimulus is detected. Tasks using this paradigm are often referred to as discrimination tasks (Figure 1B; Gilad et al., 2018; Helmchen et al., 2018) and in general take longer to learn than the simple Go/NoGo task (1–3 weeks; Guo et al., 2014a; Gilad et al., 2018; Helmchen et al., 2018). When water-deprived, animals are typically highly motivated to perform the task (Guo et al., 2014a). Since the instinctive reaction is to lick as soon as the stimuli has been detected, a short delay can be incorporated between stimulus and response period to temporally separate the different epochs (Guo et al., 2014a), however implementing a delay requires further training (Helmchen et al., 2018).

### Two Alternative Forced Choice

Similar to the Go/NoGo task, the two-alternative forced-choice (2AFC) task (Figure 1C) can be used to explore perceptual decision making. Here, an animal is presented with two stimuli however, instead of withholding a response to one of the stimuli (as in the Go/NoGo task), the animal now must report in both cases. This can be accomplished by either having the animal lick left or right (O’Connor et al., 2010a,b; Guo et al., 2014b; Peron et al., 2015; Zhong et al., 2019), turn a steering wheel (Burgess et al., 2017) or push a joystick (Estebanez et al., 2017; Morandell and Huber, 2017). The 2AFC task adds another level of clarity to behavioral outcomes, as the task design eliminates uncertainty behind NoGo responses, which may not be due to an inability to perform the task, but instead due to lack of motivation/engagement. The 2AFC task is however not completely immune to behavioral response misinterpretations, as animals can be biased toward one side of reporting (Guo et al., 2014a). As with all behaviors, the time it takes an animal to learn the 2AFC task depends on several factors such as sensory modality, type of stimulus, training methods and number of trials per session. However, due to its increased complexity compared to the simple discrimination tasks, learning the 2AFC task, in general, takes longer, ranging between 1 and 6 weeks (Mayrhofer et al., 2013; Guo et al., 2014a,b). Although it potentially can be considered a more robust behavior than the Go/NoGo tasks, the increased learning time can be a consideration for various chronic recording techniques. The 2AFC task is adaptable and can be changed according to particular experiments/questions, such as the two alternative unforced choices (2AUC) task which combines the 2AFC task with a “NoGo” signal, rewarding the animal for not responding (Burgess et al., 2017). The adaptability of the 2AFC task has led to many sensory systems being probed using this two-response behavior, including the vibrissal (Mayrhofer et al., 2013; Li et al., 2015; Peron et al., 2015), olfactory (Zariwala et al., 2013), visual (Busse et al., 2011; Burgess et al., 2017) and auditory (Znamenskiy and Zador, 2013; Wei et al., 2019) to name a few.

### Working Memory Models

To probe the neural basis of higher cognitive functions such as working memory in the head-fixed preparation, behaviors have been developed that are based on the simpler behavioral tasks, such as the Go/NoGo and 2AFC. Working memory has been addressed using a delay-task in both monkeys (Quintana et al., 1988; Yajeya et al., 1988; Fuster, 2001) and rodents (Gilad et al., 2018; Inagaki et al., 2019). Here, animals are presented with a stimulus followed by a delay during which the animal needs to retain their decision until they are able to report their decision, often indicated by a response cue (Figure 1D). Of greater complexity are the comparison-based working memory tasks such as (but not limited to) Delayed Nonmatch to Sample (Figure 1E; Dudchenko, 2004), which engages working memory by having two sensory stimuli separated by an inter-stimulus delay, forcing the animal to retain a memory trace of the first stimulus to compare to the second stimulus. For the Nonmatch to Sample working memory task the animal has to compare the stimuli and report if the second stimulus is different (mismatch) to the sample or withhold its response if the two are identical (match). This working memory task builds on the principles of the Go/NoGo task and has for example been used for odor samples (Liu et al., 2014). The comparison-based working memory tasks can also build upon the principles of the 2AFC paradigm, as is the case for the pioneering Flutter Discrimination task developed for primates (Mountcastle et al., 1990; Hernández et al., 1997). This task has been successfully adapted to freely moving rats (Fassihi et al., 2014), but to our knowledge, it has not yet been successfully adapted to head-fixed rodents. For a review on other working memory tasks in rodents, see Dudchenko (2004).

### Locomotion and Exploration

Many, if not all, behaviors require the integration of feedforward and feedback information from multiple senses. Take for example spatial exploration. Here, the visual system relies on the coordination of visual input with motor output to successfully explore and navigate through an environment (Randel et al., 2014; Heindorf et al., 2018). Probing sub-cellular neural activity during locomotion requires a behavioral platform that enables movement and associated stimuli in a head-fixed preparation. This can be achieved with a spherical (Dombeck et al., 2007; Harvey et al., 2012; Schmidt-Hieber and Häusser, 2013; Heindorf et al., 2018), circular (Hawrylycz et al., 2016; Schneider et al., 2018) or linear (Domnisoru et al., 2013; Lovett-Barron et al., 2014; Cichon and Gan, 2015; Bittner et al., 2017) track which allows precise location to be measured during locomotion in small laboratory animals. These platforms can be paired with a virtual reality-based environment. Combined with various sensory stimuli, platforms that enable locomotion during head-fixation are used to address various questions, including the neural activity underlying spatial navigation (Harvey et al., 2009; Dombeck et al., 2010; Domnisoru et al., 2013; Sheffield et al., 2017; Thurley and Ayaz, 2017), sensory processing (Niell and Stryker, 2010; Saleem et al., 2013; Makino and Komiyama, 2015; Sofroniew et al., 2015; Radvansky and Dombeck, 2018), arousal (Niell and Stryker, 2010; Polack et al., 2013; McGinley et al., 2015; Vinck et al., 2015; Shimaoka et al., 2018) and learning and memory (Lovett-Barron et al., 2014; Cichon and Gan, 2015). Behavioral platforms have also been developed that enable head-fixed mice to navigate through a physical environment (Kislin et al., 2014; Nashaat et al., 2016), as opposed to a virtual reality-based environment. Termed the air-track system (Nashaat et al., 2016), this is a lightweight maze floating on air, much like the spherical ball used in virtual reality set-ups, where the animal is head-fixed and uses its paws to navigate the maze. The air-track system offers both the stability required for most single-cell recording techniques as well as activation of different sensory modalities coupled to motor output in a “real-world” fashion.

## Exploring Cortical Functions Using Head-Fixed Behavioral Models

**Figure 2:**
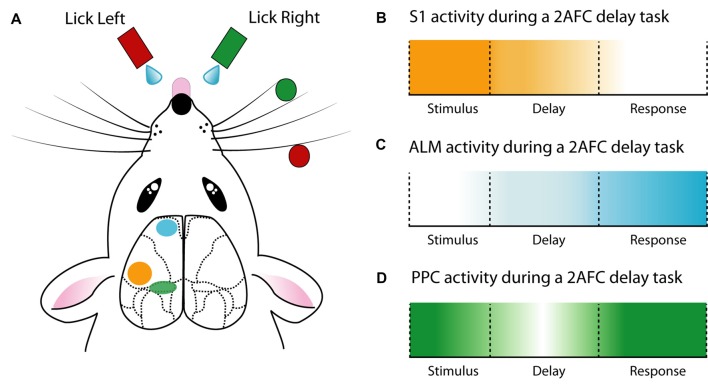
Cortical activity during a delay 2AFC task. **(A)** Schematic of a whisker-mediated object localization 2AFC task. Modified from O’Connor et al. (2010a,b), Guo et al. (2014b) and Chen et al. (2017). **(B)** The primary somatosensory cortex (S1) has maximal influence during stimulus presentation (Guo et al., 2014b). **(C)** Anterior lateral motor cortex (ALM) ramps activity during the delay epoch and reaches a maximum during the response epoch (Guo et al., 2014b; Li et al., 2015; Inagaki et al., 2018). **(D)** The posterior parietal cortex (PPC) is active throughout the 2AFC task, especially the stimulus and response epochs (Harvey et al., 2012; Goard et al., 2016).

The neocortex is enigmatic. It is a diverse structure with various regions dedicated to specific aspects of behaviors such as encoding sensory information and integrating this information with other sensory inputs. With the classical view of the role of the cortex during simple sensory-based behavior and encoding recently being challenged (Hong et al., 2018; Beltramo and Scanziani, 2019), deeper insight into the neural networks contributing to sensory perception and cognition is required. Below are summaries of the neural encoding of behavior in selected cortical regions that aim to disentangle their role in sensory-based behaviors (Figure 2).

### Primary Sensory Cortices

The role of the primary sensory cortex is to encode sensory information. To investigate the neural basis of sensory encoding at the cellular or sub-cellular level, various head-fixed behavioral paradigms have been developed. Combining the clearly defined cortical-microstructure with the prominent and easily assessable location of whiskers, the barrel cortex provides an ideal primary sense to probe during sensory-based behavior in the head-fixed preparation. Therefore, numerous studies have probed the neural activity contributing to vibrissae–based behavior. For example, the neural activity underlying perception of whisker deflection, induced by magnetic stimulation of identified whiskers coated with metallic particles has been probed using two-photon calcium imaging (Takahashi et al., 2016) and whole-cell patch-clamp (Sachidhanandam et al., 2013). Using a simple Go/NoGo behavioral task, these studies reveal that the perception of highly controlled whisker deflection requires calcium spikes in apical dendrites of layer 5 pyramidal neurons (Takahashi et al., 2016) and generates a reliable excitatory response in the somatic membrane potential which depends on cortical state (Sachidhanandam et al., 2013). Similarly, several studies have contributed to the understanding of whisker-mediated object localization, using variations of the Go/NoGo paradigm. For instance, active touch, which is the combination of whisking and touch, generates a strong activation of the apical tuft dendrites of layer 5 pyramidal neurons in the barrel cortex, driven by projections from the primary motor cortex (Xu et al., 2012). Axonal projections from the primary motor cortex to S1 were found to relay signals related to many task-related features, which, combined with ongoing sensory input, allows S1 neurons to combine and compute object localization (Petreanu et al., 2012). Moreover, S1 projections are recruited differently depending on task conditions. Chen et al. (2013) reported higher activity in neurons projecting to the primary motor cortex during the object localization task, while more neurons which project to secondary somatosensory cortex were active during texture discrimination. Despite these differences, both primary motor cortex-projecting and secondary somatosensory cortex-projecting S1 neurons could discriminate between Go and NoGo trials. Neural encoding of trial type during whisker-based object localization has also been shown to be both cell-type (Yu et al., 2019) and layer-specific, with a greater proportion of discriminating neurons located within layer 4 and 5 of the barrel cortex (O’Connor et al., 2010b). Taken together, the simple Go/NoGo behavioral task adopted in various laboratories has helped unravel the complex microcircuitry underlying touch perception and object localization.

Another sensory cortical region that has been the focus of much research is the primary visual cortex (V1). Here, the head-fixed preparation is advantageous as the animal can be oriented towards the visual stimulus (although this does not ensure stereotyped pupil location, see Wallace et al., 2013). By combining the head-fixed preparation with a spherical treadmill (Niell and Stryker, 2010) illustrated that the V1 response is strongly modulated by behavioral state, i.e., moving, stationary, sleeping, etc. Surprisingly, even in the absence of visual input, V1 neurons typically respond to a combination of running speed and visual speed (Polack et al., 2013; Saleem et al., 2013). Other studies have focused on the involvement of V1 in perceptual decision-making tasks. Here, V1 neurons were found to respond mainly during stimulus presentation, and inhibition of V1 impaired performance of the task (Goard et al., 2016). The head-fixed preparation also enables precise control over the direction and intensity of sound delivery for investigating sensory encoding and functional connectivity in the primary auditory cortex (A1; Francis et al., 2018). Using a sound-guided detection task (Kato et al., 2015) found that task engagement results in an increase in the fraction of responsive excitatory neurons, whereas responsive somatostatin expressing interneurons were reduced. Similar results were obtained in another study using two behavioral tasks of different complexity. Here, A1 can be bypassed when discriminating between simple sounds but is necessary when the discrimination involves more complex sounds with frequency overlap (Ceballo et al., 2019).

### Posterior Parietal Cortex

The posterior parietal cortex (PPC) is a classical association cortical area which is involved in various behaviors, such as decision making (Raposo et al., 2014; Goard et al., 2016; Runyan et al., 2017), evidence accumulation (Morcos and Harvey, 2016), navigation (Krumin et al., 2018) and sensory representation (Song et al., 2017; Akrami et al., 2018; Mohan et al., 2018). Often described as an integrative hub for multiple senses (Song et al., 2017; Mohan et al., 2018; Lyamzin and Benucci, 2019), the PPC receives input from various sensory cortices (Wilber et al., 2015; Zhuang et al., 2017), the thalamus (Reep et al., 1994) as well as neuromodulatory input from the basal forebrain (Broussard, 2012).

Using a memory-guided navigation task in a virtual reality setup (Harvey et al., 2012) explored the neural dynamics of L2/3 pyramidal neurons within PPC using two-photon calcium imaging. Here, neurons exhibited choice-specific sequences that could be categorized according to their activity pattern during different task epochs (epoch-specific activity). This encoding of memory-guided decisions was mostly stable on single days but was reorganized over weeks (Driscoll et al., 2017). Although overall PPC activity reaches a set point to perform the memory-guided navigation task, individual neurons shift their activity over days, some being more inconsistent than others. Learned task features may have less consistency over time, as the neurons with the least consistent relationship between activity and behavior received greater information about task features, such as trial type and maze position (Driscoll et al., 2017). Since the PPC encodes posture (Mimica et al., 2018), it is important to note that similar involvement of the PPC during memory-guided decisions was also recorded in head-fixed visual discrimination tasks that did not involve navigation (Goard et al., 2016).

### Anterior Lateral Motor Cortex

Due to the known involvement in motor planning and movement, the role of the anterior lateral motor cortex (ALM) has been extensively investigated during behavior. Building on previous work (Guo et al., 2014b) demonstrated the importance of the ALM in a 2AFC whisker-mediated delay task. Here, the ALM showed choice-specific preparatory activity as well as movement-related activity, with unilateral inhibition of the ALM during the delay epoch biasing the choice to the ipsilateral direction. Confirming and expanding on these findings (Li et al., 2015) demonstrated that ALM neurons have lateralized preference for contralateral movements. This lateralization was found to be driven by pyramidal tract neurons and not intratelencephallic neurons, although both were activated during preparatory activity. Within the ALM, the preparatory activity in intratelencephallic neurons was converted to motor output in pyramidal tract neurons, affecting the upcoming motor output. Interestingly, despite encoding sensory information (Chen et al., 2017), the ALM is required for motor planning independent of sensory modality (Inagaki et al., 2018). Here, tactile- or auditory- based tasks both displayed diverse but consistent ALM activity during a delay epoch, with behavioral performance decreasing during ALM photoinhibition. Combined with recordings during free-behavior (Erlich et al., 2011), these studies highlight the important role of the ALM in the planning and execution of motor output.

## Important Considerations When Exploring Cortical Activity Using Head-Fixed Paradigms

While head-fixation is crucial for exploring the cellular and sub-cellular basis of many behaviors, the head-fixed state can alter overall behavior. For example, head-restrained rats have far fewer whisking movements compared with freely moving behavior (Sellien et al., 2005). Likewise (Whishaw et al., 2017) illustrated that rats used alternative strategies during exploration and reach-and-grasp movement when in the head-fixed state locating objects using “touch-release-grasp” rather than sniffing. However, when directly compared, there was no difference in performance during odor discrimination in head-fixed and freely moving mice (Abraham et al., 2012). Although sensory perception may not be dramatically altered, the head-fixed preparation limits natural aspects of behavior and the ability to naturally explore and maneuver the body during a sensory-based task. Therefore, although the head-fixed preparation is advantageous in the delivery of precise and reproducible stimuli, it has the disadvantage of a non-physiological restraint of the animal. Since recordings in both the head-fixed and freely-moving preparations illustrate that the head-fixed preparation influences brain state and neural activity (Lovett-Barron et al., 2014; Chung et al., 2017; Whishaw et al., 2017), simply freeing the animal’s body to move during recordings would not mitigate the non-physiological aspect of the head-fixed preparation. However, it must be noted that when reviewing neural activity underlying sensory-based cognitive tasks, limiting feedback from other systems such as the motor (Vinck et al., 2015; Dadarlat and Stryker, 2017; Ayaz et al., 2019) and head direction (Peyrache et al., 2015) systems can be advantageous.

It is important to consider the caveats of behavioral paradigms when exploring the cortical function and it must be noted that not all behaviors can be investigated using the head-fixed preparation, such as complex social behavior that requires physical interactions. Avoiding the need for head-fixation, miniaturized head-mounted microscopes and probes (Flusberg et al., 2008; Ghosh et al., 2011; Cai et al., 2016) allow the investigation of neural activity during natural behaviors (Helmchen et al., 2001; Sawinski et al., 2009; Miyamoto and Murayama, 2016; Chung et al., 2017; Zong et al., 2017; Meyer et al., 2018; Valero and English, 2019). Further advances using wireless head-mounted microscopes (Fan et al., 2011; Pinnell et al., 2015) remove the mechanical disturbances caused by cables attached to the animal. The use of miniaturized recording techniques allows freedom of movement, which is advantageous from an overall behavioral aspect, however freely moving animals do not allow the same highly controllable settings the head-fixed preparation allows. Furthermore, not all recording techniques have been developed for the freely moving preparation, and some head-mounted probes are not suitable for mice (due to weight and size requirements). Therefore, the head-fixed preparation is still typically deemed necessary for the in-depth investigation of cortical and sub-cortical activity during many behaviors.

## Conclusion

The cortex and its role in behavior is enigmatic. To understand the underlying cortical circuits required for the execution of behaviors, it is crucial to examine neural activity while an animal is behaving. This can be achieved using the head-fixed preparation which provides highly controllable settings, and thereby precise stimuli presentation while allowing simultaneous cellular and sub-cellular recordings. The head-fixed preparation can be combined with behavioral paradigms ranging from simple sensory detection tasks, to complex, cross-modal, memory-guided decision-making tasks, allowing in-depth investigation into the neural activity underlying behaviors. Experiments using this preparation have provided insight into neural activity in various brain regions during sensory processing and higher-order cognitive functions. Although the head-fixed preparation limits natural behavior due to the head restraint, it provides valuable information about cognitive behaviors and the cortices involved. We are only beginning to understand and unravel the complexity of the neural activity underlying behavior, and while there is a rapid improvement in tethered and wireless recording techniques, the head-fixed preparation seems unlikely to be replaced as improvements to the head-fixed preparation steadily provides new platforms and setups, as well as new behavioral tasks to explore cortical functions during simple and complex behaviors. As recording techniques continue to push the boundaries of possibilities, such as functional imaging of deep brain structures with three-photon imaging (Wang et al., 2018) and simultaneous extracellular voltage recordings from hundreds of channels over 10 mm using the neuropixel probes (Jun et al., 2017), the head-fixed preparation will also continue to advance. For example, self-initiated head-fixation in the home cage will allow for the neural activity underlying more physiological behaviors to be investigated (Murphy et al., 2016). While there is no one-fits-all behavioral model, the flexibility of the head-fixed behavioral paradigms, combined with functional cellular and subcellular recordings and manipulations, provides insight into the neural dynamics underlying behaviors. Not only does this offer valuable information about single-cell dynamics, but it also expands our understanding of the individual cortices, both their individual and combined contributions to the overall behavioral output.

## Author Contributions

A-SB and LP conceptualized and wrote the manuscript.

## Conflict of Interest

The authors declare that the research was conducted in the absence of any commercial or financial relationships that could be construed as a potential conflict of interest.
